# Limitations of Electrochemical Nitrogen Oxidation toward Nitrate

**DOI:** 10.1021/acs.jpclett.2c02459

**Published:** 2022-09-21

**Authors:** Hao Wan, Alexander Bagger, Jan Rossmeisl

**Affiliations:** † Fritz Haber Institute of the Max Planck Society, 14195 Berlin, Germany; ‡ Center for High Entropy Alloy Catalysis (CHEAC), Department of Chemistry, University of Copenhagen, Universitetsparken 5, DK-2100 Copenhagen, Denmark

## Abstract

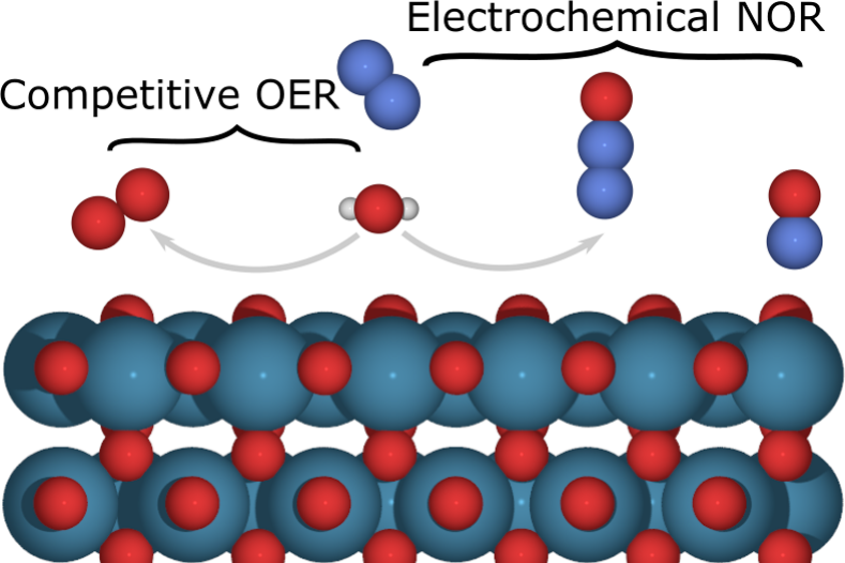

The electrocatalytic N_2_ oxidation reaction (NOR) using renewable electricity is a promising alternative to the industrial synthesis of nitrate from NH_3_ oxidation. However, breaking the triple bond in the nitrogen molecule is one of the most essential challenges in chemistry. In this work, we use density functional theory simulations to investigate the plausible reaction mechanisms of electrocatalytic NOR and its competition with oxygen evolution reaction (OER) at the atomic scale. We focus on the electrochemical conversion of inert N_2_ to active *NO during NOR. We propose formation of *N_2_O from *N_2_ and *O as the rate-determining step (RDS). Following the RDS, a microkinetic model is utilized to study the rate of NOR on metal oxides. Our results demonstrate that a lower activation energy is obtained when a catalyst binds *O weakly. We show that the reaction is extremely challenging but also that design strategies have been suggested to promote electrochemical NOR.

Nitrates are widely used as fertilizers in agriculture and oxidizing agents in explosives.^1,2^ Nitrate/nitric acid is manufactured by oxidizing ammonia using the Ostwald process, and the ammonia used here primarily comes from the Haber–Bosch process.^3^ These steps involve processes requiring high temperature (∼700 K) and high pressure (∼150 atm), leading to high energy consumption and large amounts of carbon dioxide emission from the steam reforming process.^4−6^ As a result, it is of great interest to bypass the ammonia route and develop a direct and sustainable strategy for nitrate synthesis.^2,7,8^

As a possible approach to produce nitrogen oxides and ultimately nitrate,^1,5^ direct nitrogen oxidation is, however, extremely slow at ambient conditions, and only very high temperatures or plasmas enable reasonable reaction rates.^9−11^ Electrochemical oxidative fixation of nitrogen appears to be a very attractive approach which could be driven by the electricity at ambient conditions, making the process sustainable. Figure 1a shows thermodynamic potentials at a reversible hydrogen electrode (RHE) potential scale for some important reactions, such as water oxidation and nitrogen reduction and oxidation. Even though direct N_2_ oxidation with O_2_ provides a possible solution from the thermodynamic point of view, the conflicts between O_2_ dissociation, where strong *O adsorption catalysts are needed,^12^ and N_2_ activation, which demands a weak *O adsorption catalyst,^13^ limit the O_2_ as the reactant. It can be seen that the reaction (N_2_(g) + 6H_2_O(l) → 2HNO_3_(g) + 10(H^+^ + e^–^)) has an equilibrium potential of 1.32 V vs RHE, and it has been suggested that nitrate ion production is thermodynamically favored over the competitive oxygen evolution reaction (OER) at pH above 1.3 in a wide potential region.^5^ In the past two years, a few materials, such as Pd/MXenes,^14^ several oxides (spinel oxide,^15^ Ru/TiO_2_,^16^ and PdO_2_-based^17,18^) were reported as potential electrocatalysts for NOR toward nitrate.^19−23^Figure 1b,c displays Faradaic efficiency (FE) and yields for nitrate formation from experiments. As can be observed, the FE and currents are low because of the severely competitive OER.

**Figure 1:**
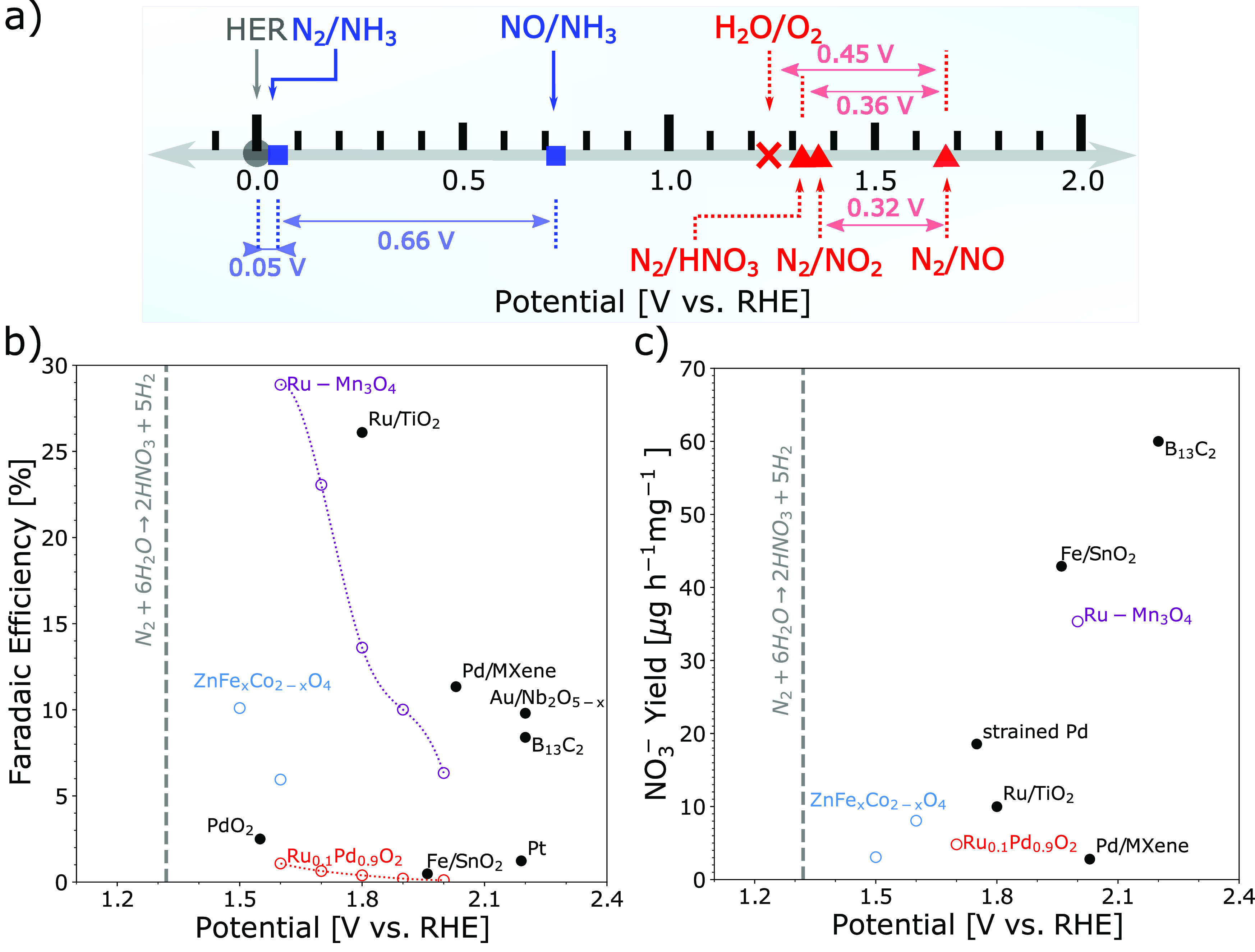
(a) Redox couples for nitrogen reduction (blue) and oxidation (red) with thermodynamic potentials. Note HNO_3_ is gas. The difference between oxygen evolution and nitrogen oxidation is shown to highlight the selectivity challenge for nitrogen oxidation. (b) Experimental reported faradaic efficiency and (c) yield of nitrate production for NOR on different materials, like Pd/MXenes,^14^ spinel oxide,^15^ Ru/TiO_2_,^16^ PdO_2_-based,^17,18^ Pt,^19^ Fe/SnO_2_,^20^ Au/Nb_2_O_5–*x*_,^21^ B_13_C_12_,^22^ and Ru–Mn_3_O4^23^ against potentials. The gray vertical line indicates the equilibrium potential of NOR toward HNO_3_.

For electrochemical NOR, it has been proposed that the nitrate formation from N_2_ oxidation can be divided into two steps: (i) the conversion of N_2_ into the *NO intermediate (* denotes the active site) and (ii) the transformation of *NO to nitrate. The former is an electrocatalytic process, which is considered as the rate-limiting step;^13^ the latter is a non-electrochemical redox reaction where the conversion of NO to HNO_3_ is known to occur readily through reaction with water.^24^ As a result, uncovering the conversion N_2_ toward *NO is needed in order to understand the electrochemical NOR.

In this study, the goal is to contribute to the understanding of the electrochemical NOR by establishing a theoretical framework. We aim to provide design strategies for NOR electrocatalysts both in terms of reaction rates and selectivity toward N_2_ oxidation relative to oxygen evolution reaction, OER. For the activation of N_2_, we evaluate different pathways and investigate the activation barriers to identify a possible rate-determining step in NOR. A classification scheme is utilized to investigate key intermediates (e.g., *N_2_O and *NO) during the activation of N_2_ among a class of metal oxide catalysts. The adsorption energy of *N_2_ and the adsorption energy difference between *O and *OH have been applied to describe the competition between OER and NOR. On this basis, we suggest the limitations of electrochemical NOR toward nitrate.

For electrochemical N_2_ activation, we consider the N_2_ triple bond to be activated via three different pathways as seen in Figure 2. (I) Dissociative path: direct dissociation of N_2_ is possible, when the metal oxides have a strong nitrogen adsorption.^25^ (II) Hydroxy path: the N_2_ is activated by a water molecule, forming *N_2_OH. (III) Oxygen path: N_2_ activation is achieved via reaction with an adsorbed *O.

**Figure 2:**
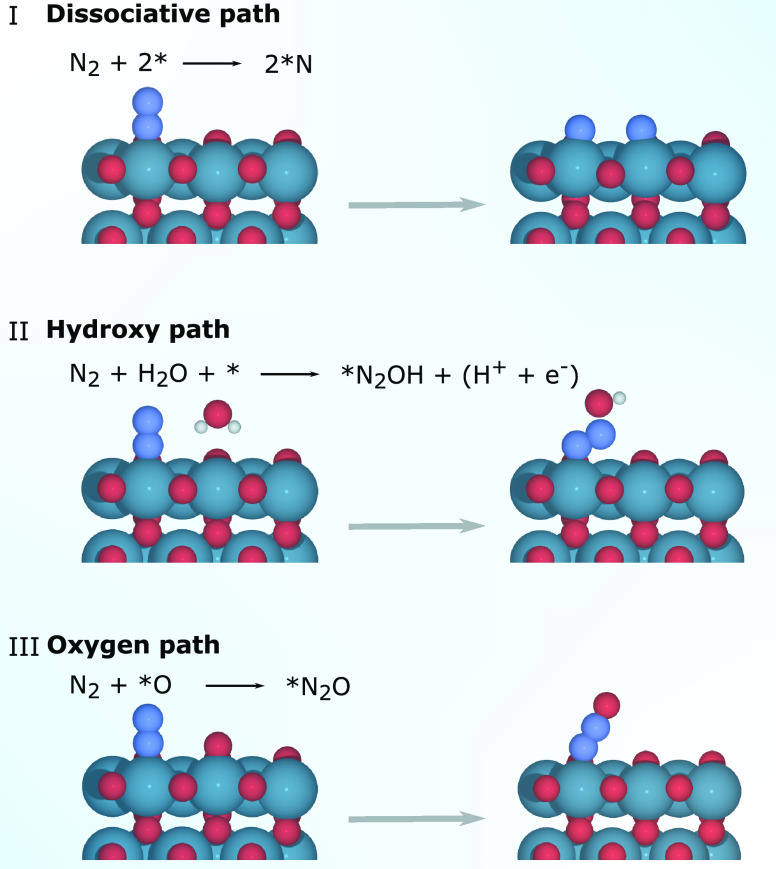
Scheme for N_2_ activation: (I) Dissociative path: N_2_(g) + 2* → 2*N; (II) Hydroxy path: N_2_(g) + H_2_O → *N_2_OH + (H^+^ + e^–^); (III) Oxygen path: N_2_(g) + *O → *N_2_O.

Figure 3 show simulations for the N_2_ activations via the three different paths: (I) dissociative path, (II) hydroxy path, and (III) oxygen path. Following (I) the dissociative path, a Brønsted–Evans–Polanyi (BEP) relation for N_2_(g) + 2* → 2*N, is obtained with a slope close to 1 as shown in Figure 3a. Metal oxides with a weaker 2*N binding demand a higher activation energy. Most metal oxides investigated here require energy above 1 eV, indicating that the direct N_2_ dissociation is unlikely. With respect to (II) the hydroxy path, Figure 3b shows that the direct hydoxy to *N_2_ from H_2_O for *N_2_OH formation is unfavorable with a thermodynamic binding above 1.6 eV, compared to *OH adsorption on metal oxides. As a comparison, N_2_ activation via adsorbed *OH: N_2_ + *OH → *N_2_OH is also considered (see Figure S1), and it has been observed that the required energy is beyond 2 eV for all metal oxides. As for (III) the oxygen path, Figure 3c shows that the activation barrier for *N_2_O formation from *N_2_ + *O scales with the *O adsorption energy with a slope close to −1. A lower energy barrier is found on metal oxides with a weaker *O adsorption energy. For example, metal oxides like SnO_2_, TiO_2_, and PdO_2_ are interesting candidates with activation energies below 1 eV. As a result, for electrochemical NOR, N_2_ might be activated via the oxygen path: N_2_(g) + *O → *N_2_O on weak oxygen binding oxides. Additionally, N_2_ activation via surface lattice oxygen (Mars Van Krevelen mechanism, Figure S2) also has been investigated where a lower driving force (more positive Gibbs free energy change) and a higher activation barrier are observed. Further NO formation from *N_2_O + *O shows a lower activation barrier (see Figure S3). Hence, the activation of N_2_ with the adsorbed *O is the rate-limiting step.

**Figure 3:**
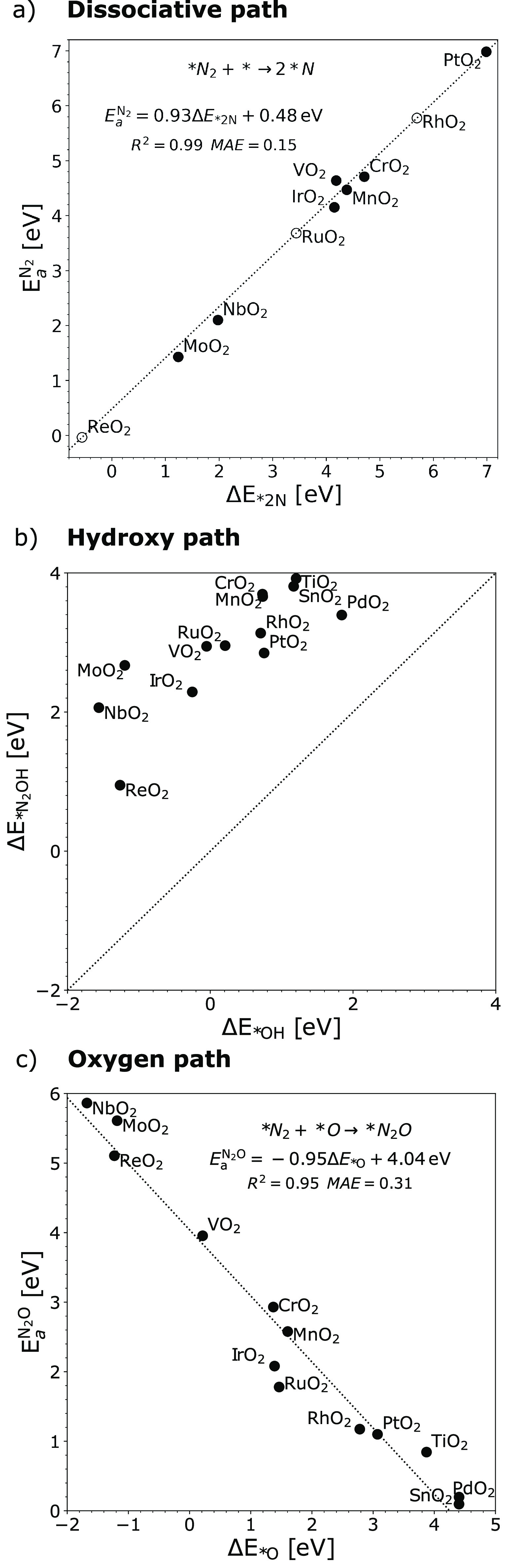
(a) Calculated transition state energy  for N_2_ dissociation as a function of dissociative chemisorption energy ΔE_*2N_. Here, unfilled markers are obtained from the linear fitting. (b) The adsorption energy of *N_2_OH plotted against the *OH adsorption energy. The diagonal line shows the equal adsorption for *OH and *N_2_OH. (c) Calculated transition state energy  for *N_2_ + *O → *N_2_O against *O adsorption energy. Considering the scaling between Δ*E* and ΔE_*O_ (Figure S4), and then  + 1.24 eV. It should be noted that N_2_ adsorption is unfavorable for most metal oxides.

As a competition for NOR, the parasitic OER has to be considered. A classification approach is utilized for understanding the competition between OER and NOR, which is similar to previous work related to CO_2_/NO/N_2_ reductions.^26−29^ First, the molecular adsorption of N_2_, N_2_O, and NO is simulated. Figure 4a shows that the *N_2_ adsorption energy is plotted against the adsorption energy of *N_2_O () on metal oxide catalysts where a close correlation between these two intermediates is observed. The horizontal dotted line demonstrates the equilibrium between N_2_(g) + * → *N_2_, while the vertical line illustrates the equilibrium between N_2_O(g) + * → *N_2_O. Three groups of catalysts can be identified: (1) both *N_2_ and *N_2_O adsorption are favorable, such as IrO_2_; (2) both *N_2_ and *N_2_O adsorption are unfavorable, like SnO_2_, TiO_2_, and PtO_2_; (3) binding *N_2_O but not *N_2_, like RuO_2_. This suggests that metal oxides in group 1, such as IrO_2_, might be capable of activating the N_2_ molecule because of its strong interaction, and it can be noted that the metal oxides which can bind the N_2_ can also bind N_2_O. Figure 4b shows the adsorption energies of *N_2_ vs *NO on metal oxide catalysts. All catalysts except SnO_2_ and TiO_2_ bind *NO. Further, the competition of OER is then considered using a microkinetic model.

**Figure 4:**
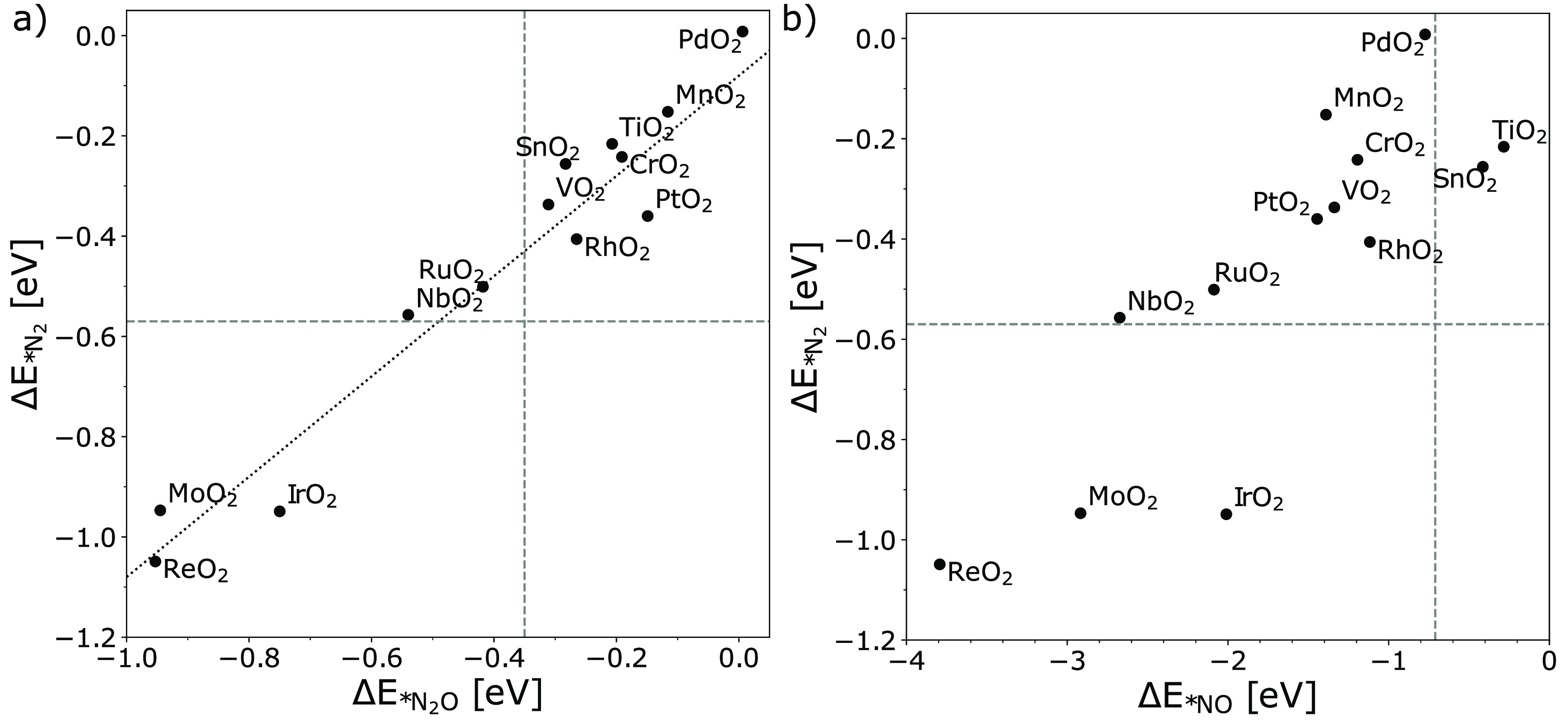
Adsorption energies of the intermediates (a) *N_2_ vs *N_2_O and (b) *N_2_ vs *NO. The horizontal lines demonstrate the equilibrium between N_2_(g) + * → *N_2_, while the vertical line in panel a illustrates the equilibrium between N_2_O(g) + * → *N_2_O and the vertical line in panel b represents the equilibrium between NO(g) + * → *NO.

One of the possible microkinetic models that can be considered for N_2_ oxidation assumes that N_2_O formation is the rate-determining step, which is suggested from Figures 3 and S3. To keep the kinetic model simple but still capturing the important chemistry, we consider the following reactions:
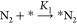
1
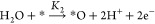
2
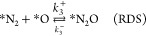
3
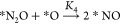
4
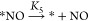
5
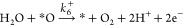
6For each step in quasi-equilibrium we can use the Langmuir isotherm:
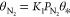
7
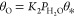
8
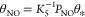
9
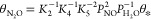
10
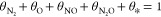
11

At low temperatures, the surface will be dominated by adsorbed *O, such that *O is the most abundant reaction intermediate, implying that θ_*_ can be written as
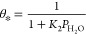
12

Assuming that eqs 1, 2, and 4 are quasi-equilibrated for NOR and that the total number of catalytic sites is fixed leads to the following analytical expression for the rate of nitrogen oxidation (*R*(NOR); for more details, see the Supporting Information). As for the rate of water oxidation (*R*(OER)) for promising NOR catalysts which can provide a reactive *O, like PtO_2_, TiO_2_, SnO_2_, and PdO_2_ (see Figure 5a), it is limited by the formation of *O (eq 2) which is potential-dependent.
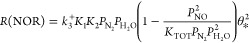
13
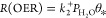
14The Faradiac efficiency (FE) for NOR is then defined by
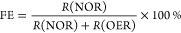
15

**Figure 5:**
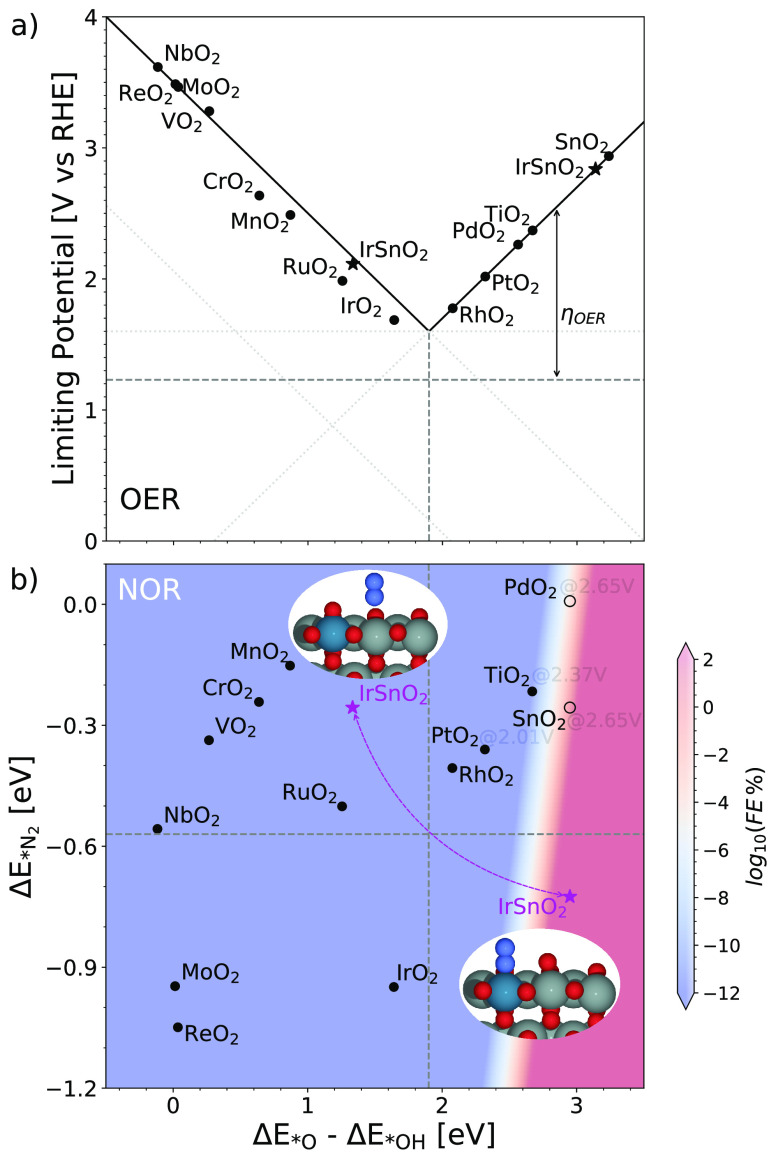
(a) OER activity volcano: the limiting potential vs ΔE_*O_ – ΔE_*OH_. The horizontal dashed line is the theoretical value (1.23 V) for OER. (b) 2-D activity heatmap describing FE (here, log_10_FE is employed) for the nitrogen oxidation as a function of (ΔE_*O_ – ΔE_*OH_) and , computed at a temperature of 300 K with (a)  = 1,  = 1. It is important to note that the coverage of *O is kept fixed by applying a potential of (ΔE_*O_ – ΔE_*OH_)/e. The vertical dotted line demonstrates the optima of ΔE_*O_ – ΔE_*OH_ for providing best OER catalytic activity. It should be noted that ΔE_*O_ – ΔE_*OH_ of SnO_2_, PdO_2_ (unfilled markers) have been adjusted using the scaling relation in Figure S6 in order to obtain simulated . Structures in panel b: IrSnO_2_. O, red; N, blue; Sn, gray; Ir, royal blue.

The reaction constant *k*_3_^+^ for eq 3 can be calculated from transition-state theory (TST) while the equilibrium constants for eq 1 (*K*_1_), eq 2 (*K*_2_), and the overall reaction (*K*_TOT_) can be computed as shown in eqs 16, 17, and 18.
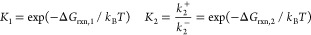
16
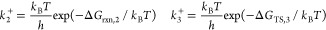
17
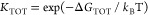
18where Δ*G*_rxn_,_1_ and Δ*G*_rxn_,_2_ are the reaction energy for eqs 1 and 2, respectively. Following the Gibbs free energy change Δ*G*_*rxn*_,_2_ for the reaction in eq 16, the rate constant *k*_2_^+^ can be expressed as *k*_2_^+^ =  where *U*_RHE_ is the applied potential, indicating that *k*_2_^+^ is potential-dependent. The rate constant, *k*_3_^+^, in eq 17 does not depend on the applied potential as this is a purely thermal heterogeneous catalytic step. The above expression for FE (eq 15) is written explicitly in terms of the pressure of the reactant (N_2_) and product (NO) relative to the standard state pressure (1 bar). In the following analysis, the influence from O_2_ partial pressure is not included, since there is a limited impact from the change of the O_2_ chemical potential under a high O_2_ partial pressure. Here, FE can be approximated using only two independent electronic energy parameters: N_2_ adsorption energy  and O adsorption energy (ΔE_*O_).

In Figure 5a, the OER activity volcano is plotted as a function of ΔE_*O_ – ΔE_*OH_. In Figure 5b, a 2-D activity heatmap also employs ΔE_*O_ – ΔE_*OH_ as a parameter, with the utilization of scaling relation between ΔE_*O_ and ΔE_*O_ – ΔE_*OH_ (see Figure S6). As a result, a 2-D activity heatmap for FE of NOR can be constructed based on these two descriptors as shown in Figure 5b where FE is computed at a temperature of 300 K with applied potential of *U*_RHE_ = (ΔG_*O_ – ΔG_*OH_)/*e* to fix *O coverage. This applied potential is also intended for adsorbed *O to be thermodynamically stable at the surface. The vertical dotted line demonstrates the optima of ΔE_*O_ – ΔE_*OH_ for providing best OER catalytic activity.

Figure 5b shows that the FE toward NO for almost all oxides is extremely low, and only SnO_2_ and PdO_2_ show limited FE for NOR under conditions of  = 1,  = 1 with temperature at 300 K. Further increasing the partial pressure for N_2_ or decreasing the content of H_2_O (see Figure S7) has only a limited improvement for the activity toward NOR, and still the OER dominates. Clearly, NOR is limited compared to OER. However, the heatmap indicates that a higher activity for NOR can be obtained when a catalyst has a stronger N_2_ adsorption and a weaker *O adsorption. As a result, mixing a weak *O adsorption catalyst like TiO_2_, PdO_2_, or SnO_2_ with strong N_2_ binding sites like Fe, Ir, and Ru could provide higher NOR activity. For example, experimentally it has been reported that Ru-doped TiO_2_ enabled a nitrate yield rate of 10.04 μg h^–1^mg^–1^ with an FE of 26.1%^16^ and Fe-SnO_2_ demonstrated a nitrate yield rate of 42.9 μg h^–1^mg^–1^ with an FE of 0.84%.^20^ Here, some bimetallic oxides, including RuTiO_2_, IrTiO_2_, IrPdO_2_, and IrSnO_2_, which are computationally constructed by the second metal atom replaced with the first metal atom, have been investigated. For example, IrSnO_2_ is constructed by the substitution of surface Sn with Ir in SnO_2_ bulk (see structures in Figure 5b). As suggested in Figure 5b, IrSnO_2_ might be an interesting candidate for NOR ( 0.34 eV, see Figure S8) when *N_2_ is adsorbed on Ir while *O sits on Sn (magenta star in red area). However, the competition might also exist if *O is adsorbed on Ir, where there is no NOR activity (the magenta star in the blue area). As for other bimetallic oxides, no NOR catalytic activity is observed because of either a weak N_2_ binding (RuTiO_2_ and IrPdO_2_) or a relatively strong *O adsorption (RuTiO_2_, IrTiO_2_, and IrPdO_2_; see Table S4).

Another more promising strategy for higher intrinsic catalytic NOR activity and selectivity is to find catalysts with a better BEP for N_2_(g) + *O → *N_2_O. The ideal BEP for this *N_2_O formation is activation energy  close to the reaction energy Δ*E*. This can be achieved by stabilizing the transition state relative to the final state. In other words, the transition state and final state should have a similar adsorption configuration, which has been employed on electrochemical O_2_ reduction by using dual-site (diporphyrin) catalysts.^12,30^ To be more specific, there is around 1.24 eV intercept difference between the BEP on metal oxides and the ideal BEP relation. On metal oxides (Figure S8), the tilting and returning process of *N_2_ during *N_2_O formation contributes to the extra barrier (1.24 eV) to overcome except the reaction energy difference part. Eliminating the tilting and returning of *N_2_ will move the BEP toward the ideal situation. This can be achieved by constructing another three-dimensional active site similar to the structure of diporphyrin to have adsorbed *O right above the adsorbed *N_2_, not like the neighboring adsorption in metal oxides. The other active site serves as an *O shuttle, which does not require the *N_2_ tilting or moving as shown in Figure S9. With the utilization of the ideal BEP relation, the stronger *O binding area is unlocked for higher NOR activity and selectivity (see Figure S10).

In this study, we use DFT simulations to investigate the possibility of the electrochemical NOR over rutile metal oxide catalysts. During the NOR process, the OER is a parasitic reaction on all metal oxides and a grand challenge to avoid. A fundamental surface catalytic limitation in terms of a compromise between selectivity and activity of NOR is identified. Similar to electrochemical N_2_ reduction, one of the challenges is really that N_2_ does not bind particularly strongly on any catalyst even though binding N_2_ on the oxides is slightly stronger than on metals.^29^ Our results propose the activation of N_2_ with *O forming *N_2_O as the rate-limiting step. Via changing the catalytic surface, it is possible to tune the reactivity of an adsorbed *O atom. This correlates with the activation energy required to activate N_2_. A less stable oxygen binding catalyst, i.e., a catalyst providing a more reactive *O, such as PdO_2_ and SnO_2_, results in a lower energy barrier to overcome for *N_2_O formation. Consequently, a higher potential needs to be applied. A 2-D activity heatmap constructed via a simple mircokinetic model demonstrates that in addition to a weaker *O adsorption, a fairly strong N_2_ adsorption might also promote a higher NOR activity. These suggest that systems mixing a weaker *O adsorption bulk like PdO_2_ and SnO_2_ with a strong N_2_ binding site, like Fe, Ru, or Ir, can be interesting candidates. These results possibly explain the experimental observation where some electrochemical NOR activity was observed on systems such as Fe-SnO_2_ and Ru-PdO_2_. Following these results, Ir-SnO_2_ has been investigated as another possible candidate for NOR when Sn has *O adsorbed and N_2_ binds on Ir. In addition, a higher N_2_ pressure and low water content also slightly promote NOR over OER. Finding electrocatalysts with a more favorable BEP for N_2_O formation can be another promising strategy for the desired NOR. These findings might benefit the way for the design and discovery of the selective and active NOR electrocatalysts. Future work for a more comprehensive investigation of bimetallic oxides might be interesting to further explore with the aim of higher selectivity. Computationally, beyond-generalized gradient approximation (GGA) approaches such GGA+U or hybrids might be interesting to be utilized to investigate the defect/polaron states in catalysts.^31^ In addition, grand canonical DFT^32−34^ could be further employed in the future to explore other possible reaction pathways and explicit dependence on pH, applied potential, surface coverages, and ions for a more detailed understanding of NOR.

## Supporting Information Available

Computational details, including different exchange correlation functionals comparison, water impact on adsorption energies, and microkinetic model construction (PDF)
