# Supplementary material for: Limitations of Electrochemical Nitrogen Oxidation toward Nitrate
Source: J Phys Chem Lett. 2022 Sep 21;13(38):8928–34. doi: 10.1021/acs.jpclett.2c02459 (PMC9531249; doi:10.1021/acs.jpclett.2c02459)
Supplement: Supplementary file 1 — jz2c02459_si_001.pdf [file jz2c02459_si_001.pdf]

Supporting Information: Limitations of Electrochemical Nitrogen Oxidation towards Nitrate

Hao Wan,*,† Alexander Bagger,‡ and Jan Rossmeisl‡

†*Fritz Haber Institute of the Max Planck Society, 14195 Berlin, Germany*

‡*Center for High Entropy Alloy Catalysis (CHEAC), Department of Chemistry, University of Copenhagen, Universitetsparken 5, DK-2100 Copenhagen, Denmark*

E-mail: wan@fhi-berlin.mpg.de

Supporting Information Available

In this supplementary information, Fig. S1 illustrates the reaction energy for $*N_2(g)$ + $*OH \rightarrow *N_2OH$ plotted against $*OH$ adsorption energy (ΔE_{*OH}). The reaction barriers for $*N_2O(g) + *O \rightarrow 2*NO$ formation is presented in Fig. S3, which includes the barriers of $*N_2(g) + *O \rightarrow *N_2O$ formation as comparison. It is demonstrated that the activation of $*N_2O$ gets easier when the adsorption of $*O$ gets weaker. In addition, a higher energy is demanded for $*N_2O$ formation compared to $2*NO$ production. Fig. S4 demonstrates that a higher driving force for $*N_2O$ formation is observed on metal oxides with a weaker $*O$ adsorption energy. The correlation between the adsorption energies of $*O$ and $*N$ is then demonstrated in Fig. S5. Fig. S5 describes the calculated adsorption energy for the $*O$ (ΔE_{*O}) plotted against the adsorption energy difference for $*O$ and $*OH$ (ΔE_{*O} - ΔE_{*OH}). Fig. S6 illustrates that 2-D activity heatmap describing FE for the nitrogen oxidation as a

function of (ΔE_{*O} - ΔE_{*OH}) and ΔE_{*N_2}, computed at a temperature of 300K. Fig. S7 shows the calculated activation energy for the reaction$N_2(g) + *O \rightarrow *N_2O$ over a $IrSnO_2$ surface. A value of 0.343 eV is found at this level of theory if $*N_2$ is adsorbed on Ir while $*O$ sits on Sn.

Computational Details

The computational analysis was carried out using the grid-based projector-augmented wave (GPAW) method, a DFT code based on a projected augmented wave (all-electron frozen core approximation) method integrated with the atomic simulation environment (ASE).[S1–S3] The revised Perdew-Burke-Ernzerhof (RPBE) functional was used as an exchange-correlation functional.[S4] The wavefunctions were represented on a uniform real-spaced grid with 0.18 Å grid-spacing and a vacuum of minimum 7 Å was employed. The unit cell of the rutile (110) metal oxide slabs consisted of four tri-layers, in total 24 metal atoms and 48 oxygen atoms corresponding to a (1×3) surface unit cell. A k-point mesh of $(3 \times 3 \times 1)$ was used to sample the Brillouin zone. Besides the calculation for climbing image nudged elastic band (NEB) was performed with GPAW code.[S5] The quasi-Newton minimization scheme was employed for the geometry optimizations, and the systems were relaxed until the forces were less than 0.05 eV/ Å. For MnO_2, spin polarized calculation has been applied while non-spin polarized calculations are conducted for other catalysts. Structures, total energies, scripts to run calculations, and plotting methods are collected in the KatlaDB database available at this link: `https://nano.ku.dk/english/research/theoretical-electrocatalysis/katladb/`.

Here, PBE + U method has been tested on SnO_2 (U = 3.5), PdO_2 (U = 7) and TiO_2 (U = 4.92) related to $*N_2$, $*O$ and $*OH$ adsorption. Following the table below, there exists a functional dependency for the intermediates adsorption energies. Especially, for PdO_2 and TiO_2, an enhancing adsorption of $*N_2$ and a weakening on energy difference between $*O$

and *OH are observed. As for the PBE + U method, there is no consistency for which value of U should use. Besides, it has been reported that the thermodynamics and the kinetics (transition state) are no longer synchronous, and the choice of U implies an error in one or the other.[S6] For now, U is generally obtained from fitting experimental band gaps. Additionally, when calculating activation energies, the U depends on where you are in the reaction path. As a result, in principle, you should use a different value of U depending on adsorption state. However, the RPBE functional[S4] level of theory has previously predicted trends in formation energy of rutile[S7] and perovskiteoxides.[S8] We therefore expected that it also correctly captured trends on adsorption energies. RPBE is employed here for all simulations.

Table S1. Functional dependence for ΔE_{*N_2}, ΔE_{*O} and ΔE_{*OH} on SnO_2, PdO_2 and TiO_2.

Species	Functional	ΔE_{*N_2}	ΔE_{*O}	ΔE_{*OH}	ΔE_{*O} - ΔE_{*OH}
SnO_2	RPBE	-0.256	1.168	4.405	3.237
SnO_2	PBE + U	-0.511	1.070	4.455	3.385
PdO_2	RPBE	0.008	1.842	4.404	2.562
PdO_2	PBE + U	-0.406	1.424	4.552	3.128
TiO_2	RPBE	-0.216	1.203	3.873	2.670
TiO_2	PBE + U	-0.511	1.106	4.215	3.109

Table S2 shows the N_2 adsorption energy comparison between RPBE and BEEF-vdW functionals. There is a slight stabilization observed from the results using BEEF-vdW functional but this adsorption energy difference is acceptable compared to DFT error (±0.1 eV) and it also does not change the results obtained from RPBE functional since most catalysts investigated here do not bind N_2 at all even including van der Waals..

Table S2. Adsorption energy difference between RPBE and BEEF-vdW for N_2 adsorption on rutile oxides.

Catalysts	IrO_2	TiO_2	PdO_2	RuO_2	MnO_2
ΔE_{*N_2}@[RPBE - BEEF-vdW]/eV	0.13	0.15	-0.05	0.16	0.16

The free energy corrections have been considered for the adsorbed oxygen evolution reaction intermediates (∗OH and ∗O), $*N_2$, $*N_2O$ and ∗NO. ΔG_{*O}, ΔG_{*OH} and ΔG_{*N_2} are employed for the mircokinetic model to obtain the rate, while thermochemical corrections for ∗O, $*N_2$, $*N_2O$ and ∗NO are utilized for obtaining the horizontal and vertical lines in Fig. 4.

To be more specific, the vibrational frequency analysis on different adsorption modes of NOR intermediates has been performed for thermochemical corrections to adsorption energy on surface. The harmonic oscillator approach implemented in ASE is utilized for catalysts where adsorbates are strongly absorbed to the surface where translations and rotations are treated as harmonic vibrations. As for adsorbates that are loosely bound to the surface such as N_2, NO and N_2O on catalysts like SnO_2, we assume negligible interaction with the surface. The adsorbates are treated as free molecules whose ΔG corrections are estimated from the reference molecules (Table S3) where translational and rotational degrees of freedom are taken into account via the the ideal-gas limit module in ASE. Corrections for ∗OH and ∗O are taken from this paper[S9] - 0.35 and 0.05 eV for ∗OH and ∗O respectively. Thus, $\Delta G_{*O} = \Delta E_{*O} + 0.05$ and likewise for ∗OH.

Table S3. Data for gas-phase H_2O, H_2, N_2 and N_2O. The data were obtained with DFT through vibrational analyses.

Species	H_2O	H_2	N_2	NO	N_2O
ZPE/eV	0.567	0.271	0.244	0.089	0.33
-TΔS/eV	-0.675	-0.407	-0.597	-0.65	-0.679

Table S4. Water impact for ΔE_{*N_2}, ΔE_{*N_2O}, ΔE_{*NO}, ΔE_{*O} and ΔE_{*OH} on PdO_2.

PdO_2	ΔE_{*N_2}	ΔE_{*N_2O}	ΔE_{*NO}	ΔE_{*O}	ΔE_{*OH}	ΔE_{*O} - ΔE_{*OH}
RPBE	0.008	0.006	-0.733	4.404	1.84	2.562
RPBE + CSM	0.031	-0.17	-0.87	4.318	1.641	2.677

As for the water influence, the continuum solvent model (CSM) has been tested for the adsorbates adsorption energies as shown in Table S4. It has been found that solvation effect

is acceptable, especially for key descriptors: ΔE_{*N2} and ΔE_{*O} - ΔE_{*OH}. In addition, almost no change in the NOR adsorbate binding energy with the inclusion of explicit water molecules is found.[S10]

Table S5. Adsorption energies for ΔE_{*N_2}, ΔE_{*O} - ΔE_{*OH} on $RuTiO_2$, $IrTiO_2$, $IrPdO_2$, and $IrSnO_2$.

Species	ΔE_{*N_2}	ΔE_{*O} - ΔE_{*OH}
$RuTiO_2$	-0.186	2.670
$IrTiO_2$	-0.662	2.670
$IrPdO_2$	-0.056	2.562
$IrSnO_2$	-0.725	2.95

A total of 10 electrons are transferred for completely oxidizing one N_2 towards HNO_3.

$N_2(g) + 6H_2O(l) \rightarrow 2HNO_3(g) + 10(H^+ + e^-)$

For N_2 triple bond activation, three different possibilities are evaluated:

I Dissociative path: N_2(g) + 2$*$ $\rightarrow$ 2$*$N;

II Hydroxy path: $N_2(g) + H_2O \rightarrow *N_2OH + (H^+ + e^-)$;

III Oxygen path: $N_2(g) + *O \rightarrow *N_2O$.

We locate the reaction barriers for I dissociative path and II hydroxy path on different rutile surfaces using the nudged elastic band (NEB) computations. The same NEB method was used to get the $*N_2O(g) + *O \rightarrow 2*NO$ formation.

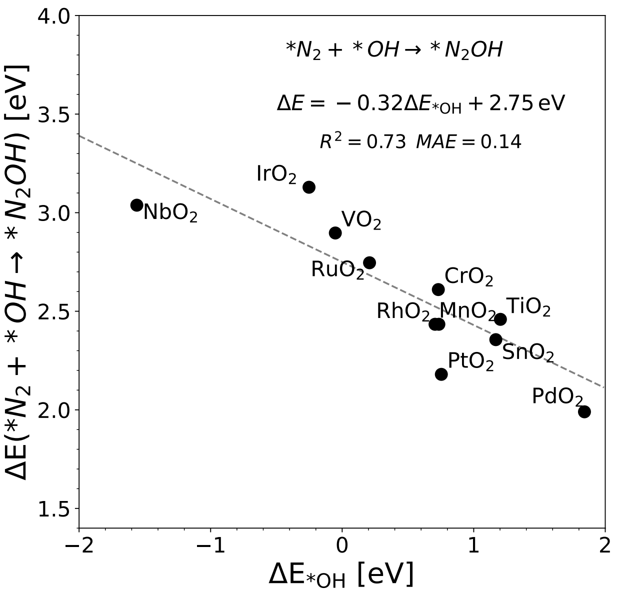

Figure S1: The adsorption energy of $*N_2OH$ plotted against the $*OH$ adsorption energy on metal oxides.

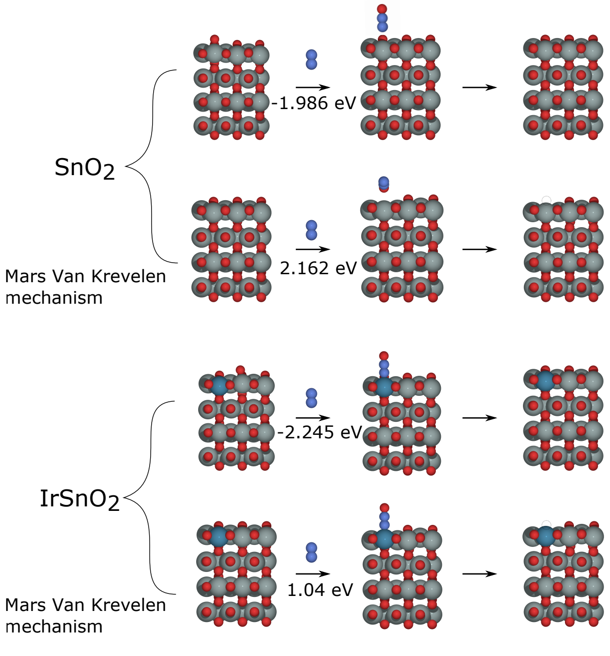

Figure S2: Reaction path for $N_2 + *O \rightarrow *N_2O$ on SnO_2 and $IrSnO_2$ with oxygen from water or lattice surface. Surface lattice oxygen (Mars Van Krevelen mechanism) also has been instigated where a lower driving force (more positive Gibbs free energy change) and a higher activation barrier are observed.

Fig. S3 shows the activation barriers for $*NO$ formation from $*N_2O$ and $*O$ intermediates plotted against O adsorption energies. Here the gray dotted line which presents N_2O formation barrier is shown as a comparison. A higher energy is demanded for N_2O formation compared to NO formation, especially on metal oxides with a strong O adsorption. This indicates that the activation of N_2 with the adsorbed $*O$ is the rate-limiting step.

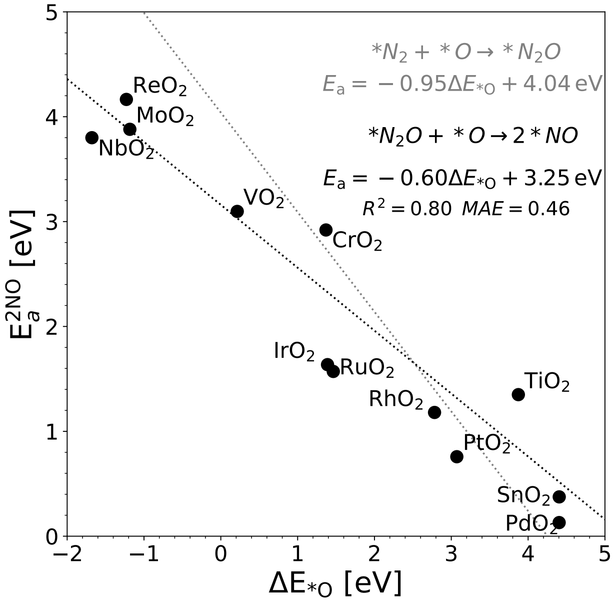

Figure S3: Scaling relationship (Brønsted-Evans-Polanyi (BEP) relation) between ΔE_{*O} and the barrier (E_a^{2NO}) for the 2 NO formation from $*N_2O$ and $*O$.

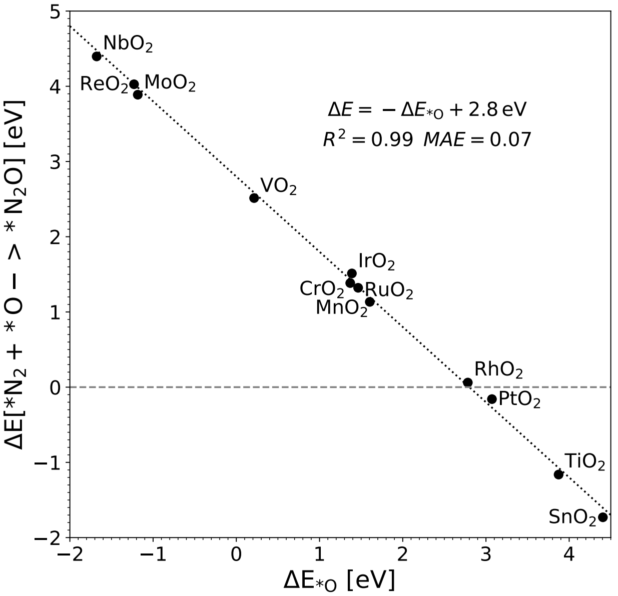

Figure S4: The correlation between the adsorption energy of $*O$ and the formation energy of $*N_2O$ from $*N_2 + *O$ on various metal oxides.

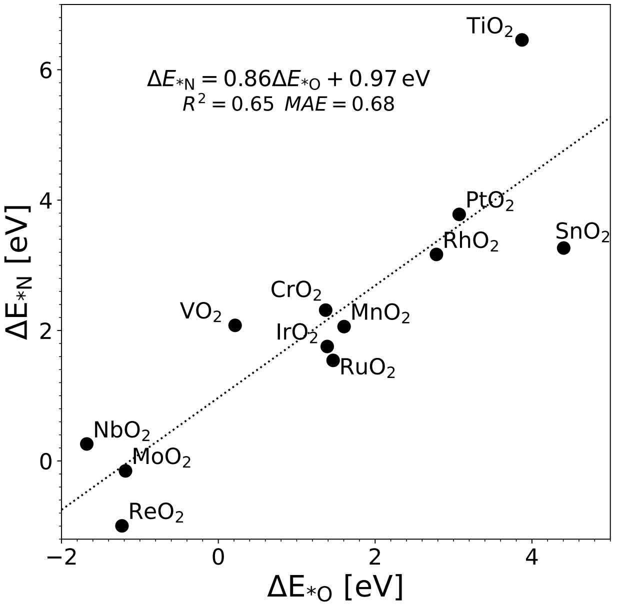

Figure S5: The adsorption energy of $*O$ is plotted versus the adsorption energy of $*N$ on various metal oxides. Metal oxide with a stronger ΔE_{*O} tends to have a stronger ΔE_{*N}.

Regarding to the weak $*O$ binding oxides, like SnO_2 and PdO_2, the data is a little bit off the correlation fitting line shown in Fig. S6. For water electrolysis, the energy difference

between $*O$ and $*OH$ adsorption is a good descriptor, while the $*O$ adoption is a better parameter for describing the activation energy for $*N_2O$, further for the FE of NOR. As a result in 2-D activity heatmap (Fig. 5 and Fig. S6), the ΔE_{*O} - ΔE_{*OH} for PdO_2 and SnO_2, 2.95 eV is employed by using the linear fitting and their $*O$ adsorption energy ($\sim$ 4.4 eV) for a better NOR activity demonstration.

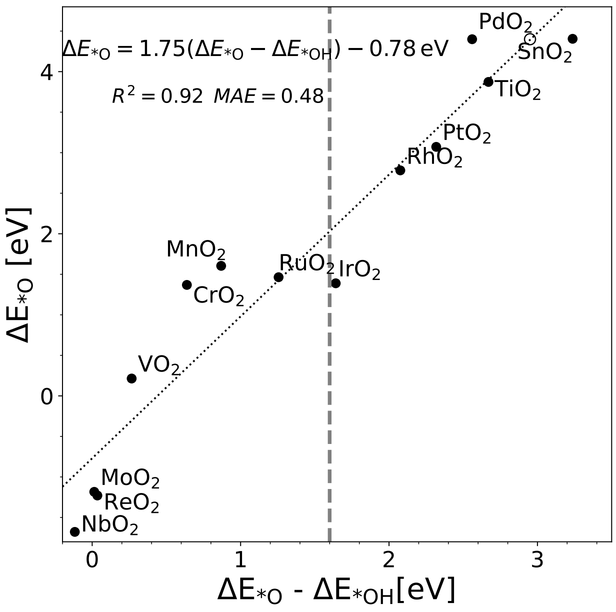

Figure S6: The correlation for the adsorption energy of $*O$ and the adsorption energy difference between $*O$ and $*OH$ intermediates. The vertical dotted line demonstrates the optima of ΔE_{*O} - ΔE_{*OH} for providing best OER catalytic activity

Microkinetic model

In this part, a more detailed derivation for the rate of NOR is provided.

$$N_2 + * \xrightarrow{K_1} * N_2 \tag{1}$$

$$H_2O + * \xrightarrow{K_2} * O + 2\,H^+ + 2\,e^- \tag{2}$$

$$* N_2 + \; * O \underset{k_3^-}{\overset{k_3^+}{\longleftrightarrow}} * N_2O \qquad (RDS) \tag{3}$$

$$* N_2O + \; * O \xrightarrow{K_4} 2 \; * NO \tag{4}$$

$$* NO \xrightarrow{K_5} * + NO \tag{5}$$

$$H_2O + \; * O \xrightarrow{k_6^+} * + O_2 + 2\,H^+ + 2\,e^- \tag{6}$$

For each step in quasi-equilibrium we can use the Langmuir isotherm:

$$\theta_{N_2} = K_1\, P_{N_2}\, \theta_* \tag{7}$$

$$\theta_O = K_2\, P_{H_2O}\, \theta_* \tag{8}$$

$$\theta_{NO} = {K_5}^{-1}\, P_{NO}\, \theta_* \tag{9}$$

Using $K_4 = \frac{k_4^+}{k_4^-}$; and $*N_2O + *O \rightarrow 2*NO$ in quasi-equilibrium, then

$$k_4{}^+\, \theta_{N_2O}\theta_O = k_4{}^-\, {\theta_{NO}}^2$$

$$\Longrightarrow \; \theta_{N_2O} = {K_4}^{-1}\, {\theta_{NO}}^2\, {\theta_O}^{-1}$$

$$\Longrightarrow \; \theta_{N_2O} = {K_4}^{-1}\, ({K_5}^{-1}\, P_{NO}\, \theta_*)^2\, (K_2\, P_{H_2O}\, \theta_*)^{-1}$$

$$\Longrightarrow \; \theta_{N_2O} = {K_2}^{-1}{K_4}^{-1}{K_5}^{-2}\, P_{NO}^2 {P_{H_2O}}^{-1}\, \theta_*$$

$$\theta_{N_2} + \theta_O + \theta_{NO} + \theta_{N_2O} + \theta_* = 1$$

For the rate-determining step, the rate of NOR (R(NOR)) is calculated in the following:

$$
\begin{aligned}
R(NOR) &= r_3{}^{+} - r_3{}^{-} \\
&= k_3{}^{+}\,\theta_{N_2}\theta_{O} - k_3{}^{-}\,\theta_{N_2O}\theta_{*} \\
&= k_3{}^{+}\,(K_1\,P_{N_2}\,\theta_*)(K_2\,P_{H_2O}\,\theta_*) - k_3{}^{-}(\,K_2{}^{-1}K_4{}^{-1}K_5{}^{-2}\,P_{NO}^{2}P_{H_2O}{}^{-1}\,\theta_*)\theta_* \\
&= k_3{}^{+}\,K_1K_2\,P_{N_2}P_{H_2O}\,(1 - K_3{}^{-1}K_1{}^{-1}\,K_2{}^{-2}K_4{}^{-1}K_5{}^{-2}\,P_{NO}^{2}P_{N_2}{}^{-1}P_{H_2O}{}^{-2})\theta_*{}^{2} \\
&= k_3{}^{+}\,K_1K_2\,P_{N_2}P_{H_2O}\,(1 - \frac{P_{NO}{}^{2}}{K_1K_2^{2}K_3\,K_4K_5{}^{2}\,P_{N_2}P_{H_2O}{}^{2}})\theta_*{}^{2} \\
&= k_3{}^{+}\,K_1K_2P_{N_2}P_{H_2O}(1 - \frac{P_{NO}{}^{2}}{K_{TOT}P_{N_2}P_{H_2O}{}^{2}})\,\theta_*{}^{2}
\end{aligned}
$$

At low temperatures, the surface will be dominated by adsorbed $*O$, such that $*O$ is the most abundant reaction intermediate, implying that θ_* can be written as:

$$\theta_* = \frac{1}{1 + K_2\,P_{H_2O}} \tag{10}$$

The $*O$ is treated as the most abundant reaction intermediate due to the following arguments. Firstly, $*O$ has been identified as the prerequisite to activate N_2 and at the same time for the interesting oxides candidates, the competitive OER is limited by the formation of $*O$. Without the formation of $*O$, both NOR and OER cannot happen. Secondly, at low temperature, with applied potential, the catalyst surface is easily covered by the OER species like $*OH$, $*O$ or $*OOH$. For a less stable oxygen binding catalyst, i.e. a catalyst providing a more reactive oxygen, such as PdO_2 and SnO_2, the potential dependent step is the formation of $*O$ or $*OH$ (see Figure 5a).

The effect from the partial pressure of N_2 (P_{N_2}) and the content of water on the faradiac efficiency of NOR is investigated. A certain promotion is observed when increasing the pressure of N_2 and/or decreasing the amount of water which both aim for suppressing the OER catalytic activity.

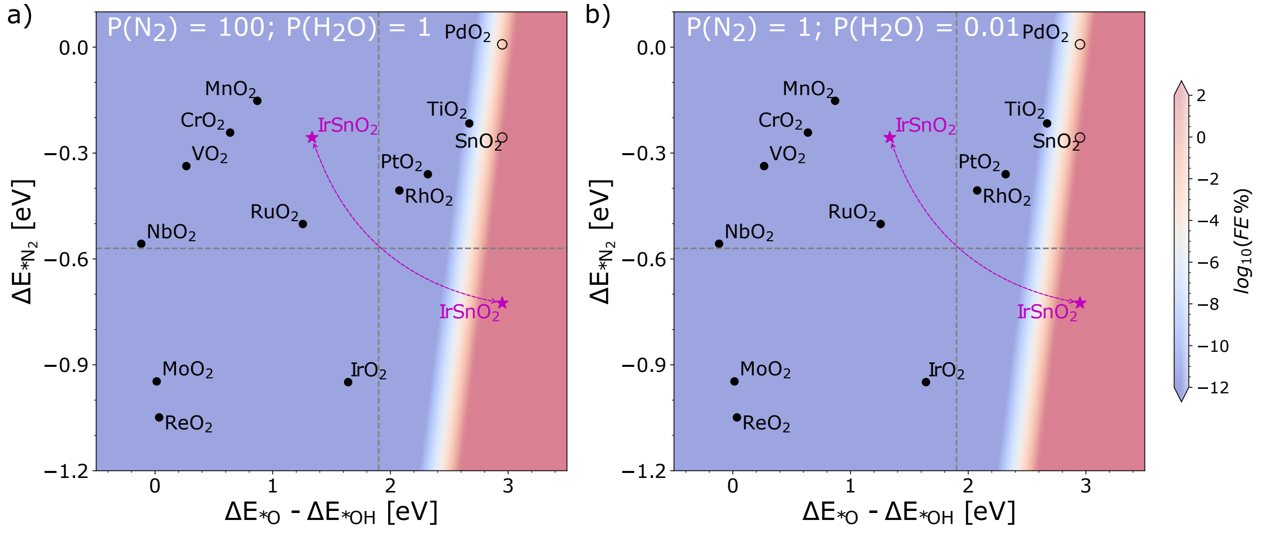

Figure S7: 2-D activity heatmap describing FE for the nitrogen oxidation as a function of (ΔE_{*O} - ΔE_{*OH}) and ΔE_{*N_2}, computed at a temperature of 300K with (a) $P_{N_2} = 100$, $P_{H_2O} = 1$; (b) $P_{N_2} = 1$, $P_{H_2O} = 0.01$. It is important to note that the coverage of $*O$ is kept fixed by applying a potential of (ΔE_{*O} - ΔE_{*OH})/e.

On metal oxides, following the microkinetic model, the NOR is determined by the reaction barrier of $*N_2 + *O \rightarrow *N_2O$. Fig. S3 shows that the reaction energy $\Delta E = \Delta E_{*O} + 2.8$ eV while $\Delta E_a{}^{N_2O}$ = -0.95ΔE_{*O} + 4.04 eV. It can be seen that $\Delta E_a{}^{N_2O} \approx \Delta E + 1.24$ eV. For $*N_2 + *O \rightarrow *N_2O$, there is a tilting of $*N_2$ towards $*O$ in the transition state (TS) and also moving back in the final state as shown in Fig. S8. This tilting and returning of $*N_2$ before and after TS contributes to the extra barrier to overcome (the intercept (1.24) of the non-ideal BEP relation).

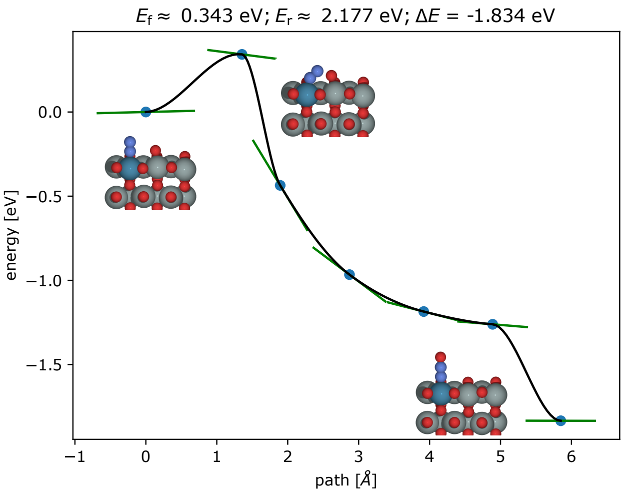

Figure S8: Reaction pathway and barrier for N_2(g) + ∗O → ∗N_2O formation on $IrSnO_2$.

In a three dimension dual-site catalyst, an another three dimension active site can have adsorbed ∗O right above the adsorbed ∗N_2 not like the neighbouring adsorption in metal oxides. The another active site serves as an ∗O shuttle, which does not require the ∗N_2 tilting or moving as shown in Fig. S8.

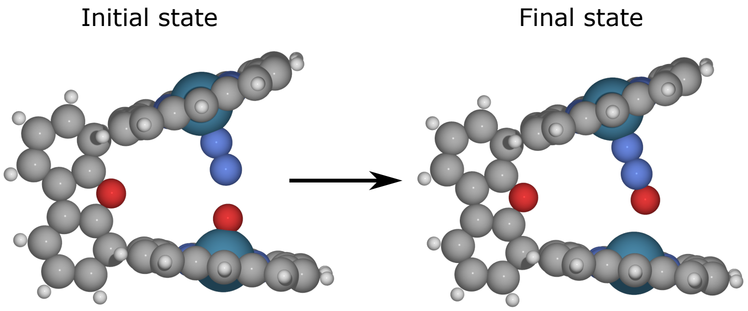

Figure S9: Illustration for N_2(g) + ∗O → ∗N_2O formation on three dimension dual-site catalyst, here using diporphyrin as an example.

The ideal BEP for ∗N_2 + ∗O → ∗N_2O is $\Delta E_a{}^{N_2O} \approx \Delta E$. With the utilization of the ideal BEP for ∗N_2O formation, a higher activity for NOR can be achieved as shown in the 2-D activity heatmap (Fig. S10). This suggests that other structures, other than metal oxides, should be investigated for finding the BEP of ∗N_2O formation close to the ideal.

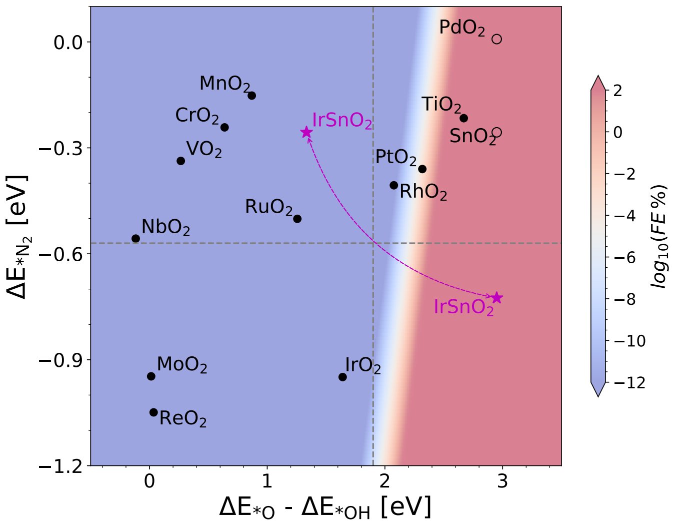

Figure S10: 2-D activity heatmap describing FE for the nitrogen oxidation as a function of (ΔE_{*O} - ΔE_{*OH}) and ΔE_{*N_2}, computed at a temperature of 300K with $P_{N_2} = 1$, $P_{H_2O} = 1$; It is important to note that the coverage of $*O$ is kept fixed by applying a potential of (ΔE_{*O}- ΔE_{*OH})/e. Here we assume that $\Delta E_a{}^{N_2O}$ in BEP relation for $*N_2 + *O \rightarrow *N_2O$ is close to ΔE ($\Delta E_a{}^{N_2O} = \Delta E = \Delta E_{*O} + 2.8$ eV see Fig. S3).

References

(S1) Mortensen, J. J.; Hansen, L. B.; Jacobsen, K. W. Real-space grid implementation of the projector augmented wave method. *Phys. Rev. B* **2005**, *71*, 035109.

(S2) Larsen, A. H.; Mortensen, J. J.; Blomqvist, J.; Castelli, I. E.; Christensen, R.; Dułak, M.; Friis, J.; Groves, M. N.; Hammer, B.; Hargus, C.; Hermes, E. D.; Jennings, P. C.; Jensen, P. B.; Kermode, J.; Kitchin, J. R.; Kolsbjerg, E. L.; Kubal, J.; Kaasbjerg, K.; Lysgaard, S.; Maronsson, J. B.; Maxson, T.; Olsen, T.; Pastewka, L.; Peterson, A.; Rostgaard, C.; Schiøtz, J.; Schütt, O.; Strange, M.; Thygesen, K. S.; Vegge, T.; Vilhelmsen, L.; Walter, M.; Zeng, Z.; Jacobsen, K. W. The atomic simulation environment—a Python library for working with atoms. *J. Phys.: Condens. Matter* **2017**, *29*, 273002.

(S3) Bahn, S. R.; Jacobsen, K. W. An object-oriented scripting interface to a legacy elec-

tronic structure code. *Comput. Sci. Eng.* **2002**, *4*, 56–66.

(S4) Hammer, B.; Hansen, L. B.; Nørskov, J. K. Improved adsorption energetics within density-functional theory using revised Perdew-Burke-Ernzerhof functionals. *Phys. Rev. B* **1999**, *59*, 7413–7421.

(S5) Henkelman, G.; Uberuaga, B. P.; Jónsson, H. A climbing image nudged elastic band method for finding saddle points and minimum energy paths. *J. Chem. Phys.* **2000**, *113*, 9901–9904.

(S6) Capdevila-Cortada, M.; Łodziana, Z.; López, N. Performance of DFT+U Approaches in the Study of Catalytic Materials. *ACS Catal.* **2016**, *6*, 8370–8379.

(S7) Calle-Vallejo, F.; Martínez, J. I.; García-Lastra, J. M.; Mogensen, M.; Rossmeisl, J. Trends in Stability of Perovskite Oxides. *Angew. Chem. Int. Ed.* **2010**, *49*, 7699–7701.

(S8) Martínez, J. I.; Hansen, H. A.; Rossmeisl, J.; Nørskov, J. K. Formation energies of rutile metal dioxides using density functional theory. *Phys. Rev. B* **2009**, *79*, 045120.

(S9) Nørskov, J. K.; Rossmeisl, J.; Logadottir, A.; Lindqvist, L.; Kitchin, J. R.; Bligaard, T.; Jónsson, H. Origin of the Overpotential for Oxygen Reduction at a Fuel-Cell Cathode. *J. Phys. Chem. B* **2004**, *108*, 17886–17892.

(S10) Anand, M.; Abraham, C. S.; Nørskov, J. K. Electrochemical oxidation of molecular nitrogen to nitric acid – towards a molecular level understanding of the challenges. *Chem. Sci.* **2021**, *12*, 6442–6448.
